# Google AdWords and Facebook Ads for Recruitment of Pregnant Women into a Prospective Cohort Study With Long-Term Follow-Up

**DOI:** 10.1007/s10995-019-02797-2

**Published:** 2019-06-20

**Authors:** Marleen M. H. J. van Gelder, Tom H. van de Belt, Lucien J. L. P. G. Engelen, Robin Hooijer, Sebastian J. H. Bredie, Nel Roeleveld

**Affiliations:** 1 Department for Health Evidence, Radboud Institute for Health Sciences, Radboud University Medical Center, Nijmegen, The Netherlands; 2 Radboud REshape Innovation Center, Radboud University Medical Center, Nijmegen, The Netherlands; 3 Department of General Internal Medicine, Radboud University Medical Center, Nijmegen, The Netherlands

**Keywords:** Epidemiologic methods, Internet, Participant recruitment, PRIDE Study, Social media

## Abstract

*Objectives* Several types of epidemiologic studies suffer from decreasing participation rates, resulting in potential selection bias and delay or termination of studies. We aimed to determine the feasibility of online methods for recruitment of pregnant women into a prospective cohort study. *Methods* In addition to traditional recruitment through prenatal care providers, we advertized participation in the PRegnancy and Infant DEvelopment (PRIDE) Study, an ongoing prospective cohort study with long-term follow-up in The Netherlands enrolling women in early pregnancy, through Google AdWords (30 days) and Facebook Ads (31 and 27 days) campaigns between September 2016 and January 2017. We calculated costs per eligible participant and compared demographics, health-related characteristics, and follow-up rates between participants recruited through online methods and prenatal care providers. *Results* During the study period, we recruited six women through AdWords (€54.28 per participant), 59 through Facebook (€10.17 per participant), and 327 through prenatal care providers (no valid cost estimate available). Facebook participants seemed to be younger (29.0 vs. 30.7 years), to have a higher body mass-index and/or low/intermediate education (27.0 vs. 24.0 kg/m^2^ and 41 vs. 25%, respectively), and to start prenatal care in secondary care more often (12 vs. 5%) than participants recruited through prenatal care providers. Item non-response and loss to follow-up rates were higher among women recruited online than among those recruited through prenatal care providers. *Conclusion* Google AdWords did not contribute substantially, but Facebook Ads may complement traditional recruitment methods of pregnant women into prospective cohort studies, despite challenges that may threaten internal validity.

**Electronic supplementary material:**

The online version of this article (10.1007/s10995-019-02797-2) contains supplementary material, which is available to authorized users.

## Significance

*What is known about this subject?* Using traditional research methods, recruitment of participants for health-related studies, including those in maternal and child health, often becomes challenging. Previous results on recruitment for cross-sectional studies through Facebook were promising, but research on recruitment for longitudinal studies and on the use of other platforms is scarce.

*What this study adds?* Facebook Ads outperformed Google AdWords for recruitment of pregnant women into a prospective cohort study with long-term follow-up and could be considered a complementary method of recruitment. Demographic characteristics including age, level of education, and BMI, and follow-up rates differed between women recruited through online methods and traditional recruitment methods.

## Introduction

Recruitment of participants for health-related research is becoming more and more challenging, with participation rates gradually declining over the last decades (Morton et al. 2006). In addition to the potential for biased results due to selective participation in particularly case–control studies, non-participation may cause delay or even early termination of studies (Gul and Ali 2010). Indeed, 25% of randomized clinical trials are discontinued, with poor recruitment being the most frequently reported reason (Kasenda et al. 2014). Cohort studies also suffer from recruitment problems: the recent termination of two large birth cohort studies, the National Children’s Study and the UK Life Study (Reardon 2014; Pearson 2015), provides an unfortunate example. Subjective experience of being busy has been identified as a reason for reduced research participation (Vercruyssen et al. 2014).

Traditionally, potential research participants have been approached by a healthcare provider, either in person or by mail or telephone, or through more general methods such as mass mailings, random digit dialing, print media, radio, and informal channels (e.g., word of mouth). With the current high access rates to the Internet, online recruitment offers an alternative to these traditional methods. A recent systematic review concluded that recruitment through Facebook should be considered when conducting health-related research, but also identified some limitations including overrepresentation of young white women (Whitaker et al. 2017). Enrollment of pregnancy planners and pregnant women in observational studies was evaluated in several previous studies (Richiardi et al. 2012; Arcia 2014; Wise et al. 2015; Admon et al. 2016; Porucznik et al. 2016; Christensen et al. 2017), but these studies either employed a cross-sectional design or retention was not evaluated. In this study, however, we assessed the feasibility of using Facebook advertisements and Google AdWords for recruitment of pregnant women into a prospective cohort study with long-term follow-up, also focusing on retention rates when the study progressed. We selected these two platforms because of their popularity among the target population (van der Veer et al. 2018; StatCounter Global Stats 2019).

## Methods

### Setting

The PRegnancy and Infant DEvelopment (PRIDE) Study is an ongoing prospective cohort study with long-term follow-up in the Netherlands aimed at identifying factors that affect the health of the women and their children at any point in time during or after pregnancy (van Gelder et al. 2013). All Dutch pregnant women aged ≥ 18 years with a gestational age < 17 weeks are eligible to participate. Data are primarily being collected through Web-based questionnaires at baseline, in gestational weeks 17 and 34, 2 and 6 months after the estimated date of delivery, and biannually throughout childhood. These questionnaires focus on demographic characteristics, obstetric history, maternal health, lifestyle factors, occupational exposures, pregnancy outcome, and infant health. Furthermore, we obtain consent for medical chart review. The PRIDE Study was approved by the Regional Committee on Research Involving Human Subjects and informed consent is obtained from all participants before inclusion in the PRIDE Study.

### Recruitment Methods

Recruitment for the PRIDE Study started in the Nijmegen region in 2011 and was gradually expanded to become nationwide in 2015. Initially, only participating midwives and gynecologists invited pregnant women for participation just before or during their first prenatal care visit (usually gestational weeks 8–12). Over 180 prenatal care providers intended to recruit participants for the PRIDE Study, but in reality, only approximately 30 midwifery practices actively invited pregnant women for participation. Because of disappointing low inclusion rates, alternative methods for recruitment of study participants were explored. For example, we exhibited the PRIDE Study at pregnancy fairs and started a collaboration with ‘Moeders voor Moeders’ (Mothers for Mothers).

As a pilot, we also advertized the PRIDE Study online through Google AdWords and Facebook Ads. For both online methods, we evaluated different search terms (based on the feedback given by the platforms) and applied search engine optimization for the study website to keep the costs per click as low as possible and to link to the search queries of potential participants. With Google AdWords, a short text advertisement that mimics the average search result on Google is shown when selected search terms are used (Online Resource). We used a set of 20 search terms related to pregnancy with a cost-per-click ranging between €0.22 and €0.45 with a maximum of €10 ($ 11.36) per day. The advertisement on Facebook was only shown to women aged 18–45 years living in The Netherlands with an interest in pregnancy. We also paid per click with a maximum of €10 per day. The costs per click were based on an automated bidding system (lowest cost bid strategy), which is the default setting in Facebook Ads (Facebook Business 2019). The advertisements used are shown in the Online Resource. After clicking on the advertisement, the person was redirected to the PRIDE Study website for additional information. Through this website, women could also sign up for participation. We aimed to display advertisement campaigns for 1 month: the AdWords campaign ran for 30 days in September–October 2016, the Facebook campaign for 31 days and 27 days in October–November 2016 and January 2017, respectively. The online recruitment strategies were staggered to be able to evaluate recruitment through Google AdWords and Facebook Ads separately.

When signing up for participation, women indicated how they learned about the PRIDE Study. For the current study, we included all participants who were recruited through their prenatal care provider, Google AdWords, or Facebook Ads between September 19, 2016 and January 31, 2017 with follow-up until November 30, 2017. Follow-up methods were identical for the different recruitment methods and did not change over the study period.

### Statistical Analysis

For the online recruitment methods, we extracted the number of total impressions (times the advertisement was shown on the website), total clicks, click-through rates (number of clicks divided by number of impressions), costs per click, and total costs from Google AdWords and Facebook Ads. The average numbers of impressions and clicks per day were obtained by dividing the total impressions and total clicks by the number of campaign days. We calculated the costs per enrolled participant by dividing the total costs per online recruitment method by the number of participants recruited through that method.

We compared participants recruited through prenatal care providers with women recruited through online recruitment methods for age at enrollment, country of birth, educational level, pre-pregnancy body mass index (BMI) based on self-reported height and weight, gravidity, parity, smoking and alcohol consumption during pregnancy, type of prenatal care provider, and gestational age at enrollment. Furthermore, we compared pregnancy outcome between these groups of participants, with missing values as a proxy for loss to follow-up. These data were obtained from the PRIDE Study questionnaires administered during pregnancy (miscarriage/stillbirth or termination of pregnancy) and 2 months after the estimated date of delivery (live-born infant or stillbirth). Consequently, all women who did not complete the post-partum questionnaire and did not report an adverse pregnancy outcome in the prenatal questionnaires were considered lost to follow-up. All statistical analyses were performed using IBM SPSS Statistics for Windows, Version 22 (IBM Corp., Armonk, NY).

## Results

A total of 327 participants were recruited through prenatal care providers during the study period. The Google AdWords campaign generated 34,325 impressions and 865 clicks (click-through rate 2.52%); total costs were €325.66 (Table 1). A total of 13 women approached through Google AdWords signed up for participation, but only six provided informed consent and were eligible for participation. The Facebook advertisement in October–November 2016 yielded 4757 clicks after 128,485 impressions (click-through rate 3.70%; total costs €315.52), with 57 women signing up for participation and 29 eligible participants. The second Facebook Ads campaign in January 2017 generated 131,142 impressions and 5244 clicks (click-through rate 4.00%, total costs €284.48), resulting in 59 women signing up for participation and 30 eligible participants. Of the 19 women ineligible for participation in the 3 campaigns combined, 18 had a gestational age ≥ 17 weeks and 1 was < 18 years of age. The costs per eligible participant for Google AdWords and Facebook Ads were €54.28 ($61.67) and €10.17 ($11.55), respectively.

**Table 1:** Recruitment results for a prospective cohort study among pregnant women using Google AdWords and Facebook Ads

	Google AdWords	Facebook (1)	Facebook (2)
Time period (days)	Sept 19–Oct 18, 2016 (30)	Oct 24–Nov 23, 2016 (31)	Jan 2–Jan 30, 2017 (27)^a^
Total costs (€)	325.66	315.52	284.48
Total impressions	34,325	128,485	131,142
Average impressions per day	1144	4145	4857
Total clicks	865	4757	5244
Average number of clicks per day	28.8	153.5	194.2
Click-through rate (%)	2.52	3.70	4.00
Costs per click (€)	0.38	0.07	0.05
Signed up for participation	13	57	59
Provided informed consent	8	41	36
Ineligible^b^	2	11	6
Participates in study	6	29^c^	30
Costs per eligible participant (€)	54.28	10.88	9.48

^a^Advertisement was offline for 2 days due to problem with budget settings

^b^Of the 19 women ineligible for participation, 18 had a gestational age ≥ 17 weeks and 1 was < 18 years of age

^c^One woman provided informed consent, but did not complete any questions in the questionnaire

The characteristics of the PRIDE Study participants recruited through prenatal care providers and Facebook are shown in Table 2. The six women recruited via Google AdWords were not included in this comparison. Although based on small numbers, participants recruited through Facebook seemed to be slightly younger (29.0 vs. 30.7 years), and had a higher pre-pregnancy BMI (27.0 vs. 24.0 kg/m^2^), whereas larger proportions seemed to have low/intermediate education (41 vs. 25%) and to start prenatal care in a secondary care setting (12 vs. 5%) compared to participants recruited through prenatal care providers. We did not observe substantial differences (> 5%) in the other demographic and health-related characteristics between the participants recruited through prenatal care providers and participants recruited through Facebook.

**Table 2:** Percentages or means (standard deviations) of characteristics of PRIDE study participants by recruitment method

Characteristic	Prenatal care provider (N = 327)	Facebook Ads (N = 59)
Maternal age (years)	30.7 (3.5)	29.0 (3.4)
Maternal country of birth (%)		
The Netherlands	92	88
Other	3	2
Missing	5	10
Maternal level of education (%)^a^		
Low/intermediate	25	41
High	71	49
Missing	4	10
Pre-pregnancy BMI (kg/m^2^)	24.0 (3.6)	27.0 (5.5)
Gravidity (%)		
0 previous pregnancies	37	39
≥ 1 previous pregnancies	62	61
Missing	0	0
Parity (%)		
0 previous births	51	54
≥ 1 previous births	49	46
Missing	0	0
Smoking during pregnancy (%)		
Yes	3	5
No	93	85
Missing	4	10
Alcohol consumption during pregnancy (%)		
Yes	8	12
No	88	78
Missing	4	10
Prenatal care provider (%)		
Midwife/general practitioner	95	88
Gynecologist	5	12
Gestational age at inclusion (weeks)	10.1 (2.7)	9.6 (3.7)
Pregnancy outcome (%)		
Live-born infant	73	61
Miscarriage/stillbirth	1	3
Termination of pregnancy	1	2
Missing	25	34

^a^High level of education: completed higher vocational education or university

Compared to women recruited through prenatal care providers, however, women recruited through Facebook more often did not complete the questions on country of birth, level of education, and smoking and alcohol consumption during pregnancy. Furthermore, participants recruited through both online methods were more often lost to follow-up compared to women recruited through prenatal care providers, as reflected in the higher rates of missing information on pregnancy outcome for Google AdWords (50%) and Facebook (34%) than for prenatal care providers (25%).

## Discussion

Our findings indicate that Facebook Ads outperform Google AdWords for recruiting pregnant women into a prospective cohort study with long-term follow-up with regard to the total number of participants enrolled and the costs per eligible participant. Participants recruited through Facebook seemed to be slightly younger, more likely to have a lower level of education and a higher pre-pregnancy BMI, and more likely to start prenatal care in a secondary care setting than women recruited through prenatal care providers. However, item non-response and loss to follow-up rates were higher among women recruited through online methods compared to recruitment through prenatal care providers.

To the best of our knowledge, this study is the first attempt to evaluate recruitment of pregnant women for research purposes using Google AdWords. Therefore, we can only hypothesize about the reasons why this online method did not work as well as Facebook Ads. Most likely, reaching the target population is much harder with Google AdWords, as it is impossible to use demographic characteristics and other background information Facebook collected from its users to select the people to which the advertisement is being shown (targeted approach). Recruitment of pregnancy planners and pregnant women into observational studies through Facebook, however, was evaluated in several previous studies with mixed results: click-through rates ranged between 0.03 and 2.74% (current study: 3.85%), whereas the costs per recruited woman ranged between €3.44 ($3.91) and $33.31 (€29.32) (current study: €10.17) (Richiardi et al. 2012; Arcia 2014; Wise et al. 2015; Admon et al. 2016; Porucznik et al. 2016; Christensen et al. 2017). Also, the number of women recruited through this social media platform differed strongly depending on the budget spent. Assuming a continuous (linear) flow of participants recruited through Facebook with a budget of €10 per day, this method may still have yielded fewer participants (N = 137) compared with recruitment through prenatal care providers (N = 327) over the study period of 135 days. Increasing the daily budget for Facebook Ads may boost the number of participants to a level similar to or exceeding healthcare provider-based recruitment, but no studies on the optimal budget settings have been performed yet.

As study participants in general and hard-to-reach subgroups, such as pregnant women, in particular are challenging to recruit nowadays, targeted Facebook advertisements seem to gain popularity as a new method of recruitment for both observational (Arcia 2014; Harris et al. 2015; Admon et al. 2016; Christensen et al. 2017; Fonseca and Canavarro 2017; Motoki et al. 2017; Jordan et al. 2018; Kerns et al. 2018; Mengesha et al. 2018) and experimental studies (Adam et al. 2016; Laws et al. 2016) within maternal and child health research. In other research areas, for example studies among men who have sex with men (Lachowsky et al. 2016; Buckingham et al. 2017), parents (Tustin et al. 2017; Oesterle et al. 2018), and cancer survivors (Juraschek et al. 2018; Tsai et al. 2019), online recruitment methods are also being applied successfully. However, Williamson et al. (2018) failed to recruit pregnant women with asthma through Facebook and Twitter and pointed to the possibility of publication bias in the literature on online recruitment methods.

Similarly to previous studies on the recruitment of pregnancy planners or pregnant women through Facebook, online recruitment for the PRIDE Study yielded a more diverse sample of pregnant women compared to healthcare provider-based recruitment, particularly with regard to maternal age (Arcia 2014), level of education (Admon et al. 2016), and gestational age at enrollment (Adam et al. 2016; Admon et al. 2016). Although these differing characteristics may yield advantages, online recruitment has also been criticized as the characteristics of women with and without Internet or Facebook in particular may differ. However, previous studies showed that this form of self-selection does not necessarily lead to biased measures of associations in prospective cohort studies (Nohr et al. 2006; Nilsen et al. 2009; Hatch et al. 2016).

We identified only two studies that reported on retention rates after online recruitment, varying between 43% at 6–8 weeks follow-up for Australian parents and 78% at 1 month follow-up for young veterans (Pedersen et al. 2017; Bennetts et al. 2019). In our study, retention seemed lower among women recruited through Facebook (66%) than among women recruited through prenatal care providers (75%), which may lead to selection bias caused by differential loss to follow-up (informative censoring) (Hernán et al. 2004). This issue represents a threat to the internal validity of effect estimates derived from cohort studies. Although statistical techniques, such as inverse probability-of-censoring weighted estimation and stratification-based methods can correct for selection bias due to loss to follow-up (Howe et al. 2016), methods to improve retention in Web-based cohorts should be developed to prevent this type of bias, irrespective of the method of recruitment.

Limitations of the current study include the relatively small numbers of subjects enrolled through online methods, the difference in time period between the three methods of recruitment, and lack of a valid cost estimate for participants recruited through prenatal care providers as we do not have insight into the number of study leaflets (€0.05 each) distributed during the study period. Based on a previous study (Bonde et al. 1998), we estimated that it takes 120 study leaflets to include one participant (€6.00 per participant).

In conclusion, recruitment of women early in pregnancy into a prospective cohort study with long-term follow-up through Facebook Ads is feasible and may improve participation among typically underrepresented populations, such as women with a low level of education. However, online recruitment also results in lower follow-up rates compared to recruitment through prenatal care providers. Google AdWords did not contribute substantially to the recruitment of pregnant women into the PRIDE Study.

## Electronic supplementary material

Below is the link to the electronic supplementary material.

Supplementary material 1 (PDF 316 kb)
