# Supplementary material for: Google AdWords and Facebook Ads for Recruitment of Pregnant Women into a Prospective Cohort Study With Long-Term Follow-Up
Source: Matern Child Health J. 2019 Jun 20;23(10):1285–91. doi: 10.1007/s10995-019-02797-2 (PMC6732125; doi:10.1007/s10995-019-02797-2)

eAppendix – Advertisements used for PRIDE Study recruitment

Google AdWords - Desktop

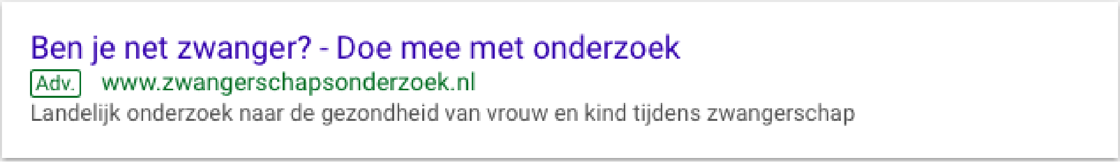

Google AdWords - Mobile

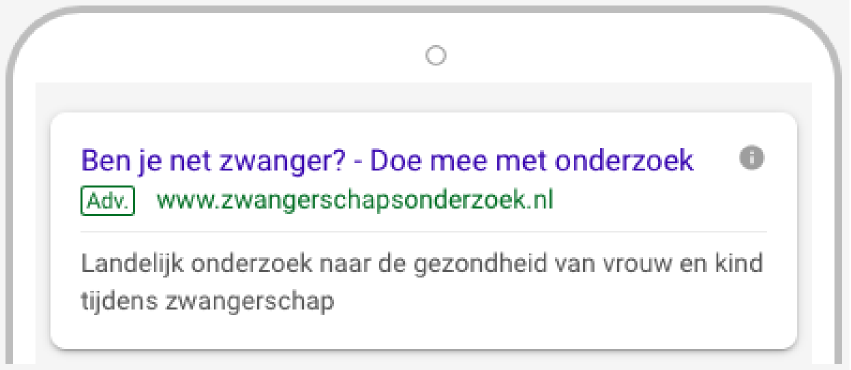

Facebook Ads - Desktop

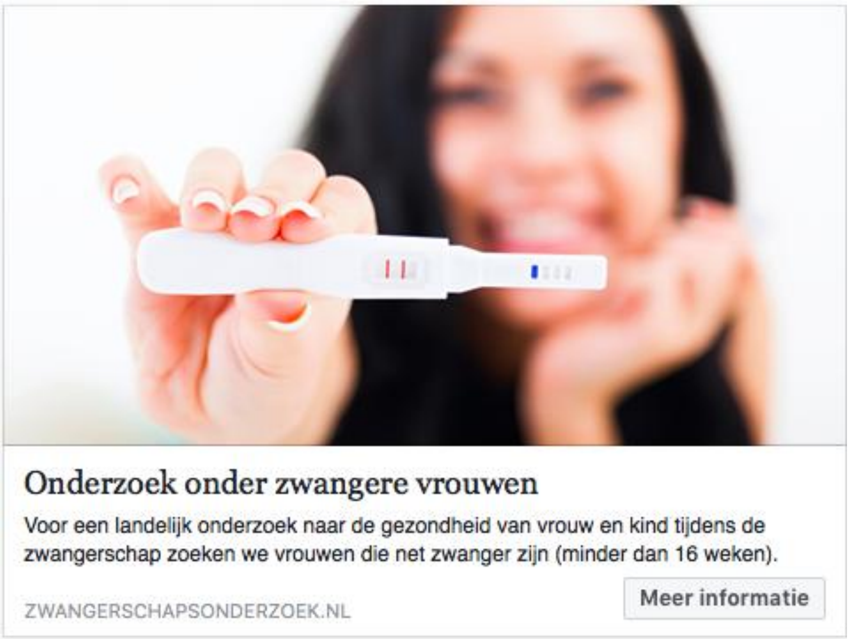

Facebook Ads - Mobile

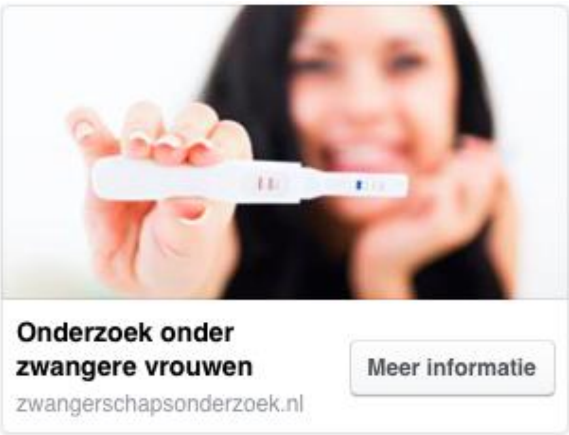

Supplement: Supplementary file 1 — Supplementary material 1 (PDF 316 kb) [file 10995_2019_2797_MOESM1_ESM.pdf]
